# Spatio-temporal dengue risk modelling in the south of Thailand: a Bayesian approach to dengue vulnerability

**DOI:** 10.7717/peerj.15619

**Published:** 2023-07-14

**Authors:** Fatima Ibrahim Abdulsalam, Pablo Antúnez, Warit Jawjit

**Affiliations:** 1 School of Public Health, Walailak University, Thasala, Nakhon Si Thammarat, Thailand; 2 División de Estudios de Postgrado, Universidad de la Sierra Juárez, Ixtlán de Juárez, Oaxaca, México

**Keywords:** Bayesian inference, CARBayesST, Climate variables, Dengue risk, Nakhon Si Thammarat, Spatio-temporal modelling

## Abstract

**Background:**

More than half of the global population is predicted to be living in areas susceptible to dengue transmission with the vast majority in Asia. Dengue fever is of public health concern, particularly in the southern region of Thailand due to favourable environmental factors for its spread. The risk of dengue infection at the population level varies in time and space among sub-populations thus, it is important to study the risk of infection considering spatio-temporal variation.

**Methods:**

This study presents a joint spatio-temporal epidemiological model in a Bayesian setting using Markov chain Monte Carlo (MCMC) simulation with the CARBayesST package of R software. For this purpose, monthly dengue records by district from 2002 to 2018 from the southern region of Thailand provided by the Ministry of Public Health of Thailand and eight environmental variables were used.

**Results:**

Results show that an increasing level of temperature, number of rainy days and sea level pressure are associated with a higher occurrence of dengue fever and consequently higher incidence risk, while an increasing level of wind speed seems to suggest a protective factor. Likewise, we found that the elevated risks of dengue in the immediate future are in the districts of Phipun, Phrom Kili, Lan Saka, Phra Phrom and Chaloem Phakiat. The resulting estimates provide insights into the effects of covariate risk factors, spatio-temporal trends and dengue-related health inequalities at the district level in southern Thailand.

**Conclusion:**

Possible implications are discussed considering some anthropogenic factors that could inhibit or enhance dengue occurrence. Risk maps indicated which districts are above and below baseline risk, allowing for the identification of local anomalies and high-risk boundaries. In the event of near future, the threat of elevated disease risk needs to be prevented and controlled considering the factors underlying the spread of mosquitoes in the Southeast Asian region.

## Introduction

Dengue fever (DF) transmission continues to occur in about half of the world’s population, particularly in people living in environmentally suitable areas (Messina et al., 2019), as the *Aedes* mosquito vector thrives best in subtropical and tropical urban cities around the world (Kraemer et al., 2015), and dengue could increase its spread and intensity due to expected climate change (Cromar & Cromar, 2021). With an estimated number of 100 million symptomatic infections and 10,000 deaths per year in over 125 countries (Stanaway et al., 2016), dengue is known to be the greatest human disease burden of an arthropod-borne virus. In endemic areas, dengue has intensified with the global warming trend resulting in a greater number of human infections expected to be severe and longer transmission seasons (Gubler, 2011; Murray, Quam & Wilder-Smith, 2013). In currently dengue-free or low-risk parts of Europe, North America, and Africa, rising temperatures attributed to climate change may further exacerbate dengue spread and transmission (Senior, 2008). The predominant dengue fever mosquito vector*, Aedes aegypti* also transmits yellow fever, chikungunya and the most recent Zika virus pandemic (Musso, Cao-Lormeau & Gubler, 2015).

The appearance and evolution of dengue in space and time vary from region to region which is directly linked, among other factors, to climate variation and anthropogenic factors (Kearney et al., 2009; Colón-González, Lake & Bentham, 2011). Currently, a vast majority of dengue transmission occurs in Asia (Messina et al., 2019) and for the past decades, DF has been of public health concern, particularly in the southern region of Thailand (Abdulsalam et al., 2021). Therefore, it is important to have the most detailed information possible on spatial and temporal patterns of behaviour to plan mitigation actions, including risk maps that allow the identification of local anomalies and high-risk boundaries. Several analysis methods have been tested, including models whose approaches consider spatial and temporal autocorrelation such as the Integrated Nested Laplace Approximations (INLA) (Rue, Martino & Chopin, 2009) and Markov chain Monte Carlo (MCMC) (Robert, 2009; Robert, Casella & Casella, 2010) which have yielded satisfactory results in previous studies (Rushworth, Lee & Mitchell, 2014; Jaya & Folmer, 2021).

Due to the severe burden of dengue infection and its relationship with weather variables, environmental factors such as rainfall, temperature and relative humidity have often been used in statistical and mathematical models; but in this study, additional weather variables like wind speed, pan-evaporation, cloud cover, sea-level pressure and number of rainy days were included. Existing studies have a different focus on dengue epidemics in the region eluding the dengue spatiotemporal model. For planning and prevention of recurrent dengue epidemics, disease modelling maps that identify spatiotemporal patterns, provide reliable estimates of relative risks, and detect high-risk areas for further investigation of the disease distribution are needed.

The main objectives were to assess environmental risk factors of dengue at the district level and evaluate areas of highest risks and their changes over time using monthly data of reported dengue cases at the district level between January 2002, to December 2018. To achieve the objectives, we formulated the following four questions which served as the guiding thread of the study: Is dengue infection risk increasing or decreasing over time?, to what extent do environmental factors affect the risk of dengue?, which areas have the highest disease risk for targeted interventions? and, is dengue-related health inequality changing over time in the Nakhon Si Thammarat province of Thailand? The results reported here constitute an advance in the knowledge of the spatiotemporal dispersion of dengue in the Nakhon Si Thammarat region, which could be useful for decision-makers not only for the region studied but also in other places with similar environmental conditions.

## Materials and Methods

### Study area

**Figure 1:**
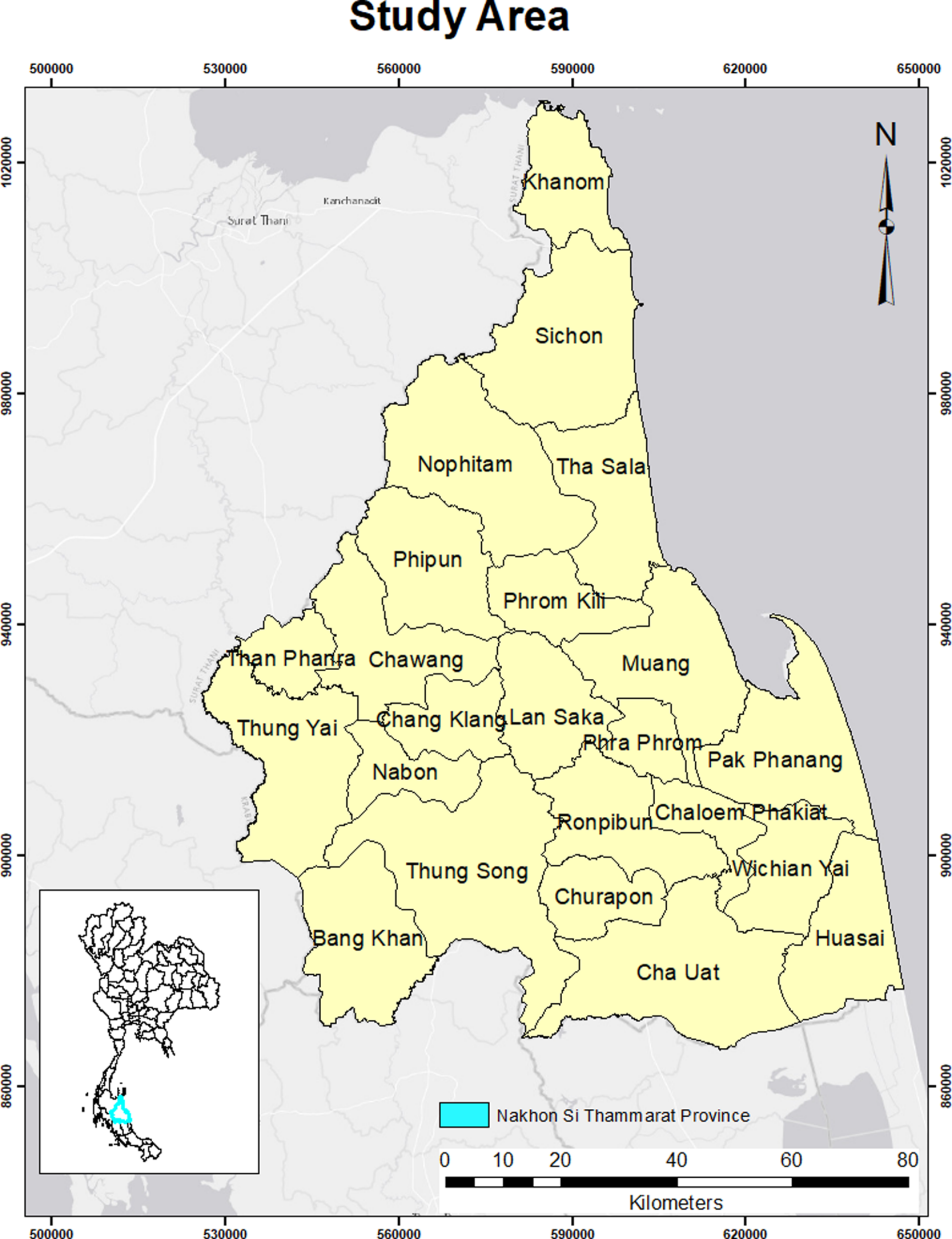
The study area shows all 23 districts of Nakhon Si Thammarat Province. Source: Natural Earth (http://www.naturalearthdata.com/).

The study area is the Nakhon Si Thammarat region located on the shore of the Gulf of Thailand (Fig. 1). With a current population of about 1,550,720 (765,369 males and 785,351 females), it has a total area of approximately 9,943 km^2^ and a population density of 156 persons/km^2^ (Provincial Office, 2021; Thailand Interior Ministry, 2021). Primarily rural, the province is divided into 23 districts and 165 sub-districts; however, the capital city called Mueang is the most densely populated area (Abdulsalam et al., 2022). The region has an average annual temperature and precipitation of 26.7 °C, and 1,978 mm respectively (ClimateData.org, 2015).

### Obtaining health data

The health data included reported monthly cases of a confirmed dengue fever diagnosis from January 2002 to December 2018 registered in the national disease surveillance report system of the Bureau of Vector-Borne Diseases (currently known as Division of Vector-Borne Disease, DVBD), Ministry of Public Health, Thailand (Bureau of Epidemiology, 2019). The R506 national surveillance system (which is similar to the national electronic disease surveillance system of the United States CDC), has health data inputs obtained from public hospitals/health centres and coded as the ICD-10 (International Statistical Classification of Disease and Health-Related Problems 10 codes), compatible with disease prevention and epidemiological studies (Thaivbd, 2019). According to clinical criteria required to be reported to the surveillance system by public hospitals and clinics every week, the Thailand R506 national surveillance system identifies dengue cases based on regulations by the world health organization (WHO, 1997). If there is a presence of acute fever with at least two clinical symptoms such as severe retro-orbital eye pain, muscle pain, headache, high fever, positive tourniquet test, or a leukocyte count <5,000/µL, a confirmed case of dengue fever is recorded (Ministry of Public Health, 2019). Also, a case of dengue haemorrhagic fever is defined by a haematocrit elevation of 10–20%, and serology confirmation of all reported cases varies between 10% and 50% (Ministry of Public Health, 2019). Despite the effort to keep a realistic record, the real outcome of dengue fever cases could be slightly underestimated due to considerable uncertainty of self-reported data (Abdulsalam et al., 2021). However, we assume that it is of sufficient quality to make inferences without significant bias as the degree to which patients seek medical attention may be affected by the severity and duration of symptoms.

### Obtaining environmental variables

The environmental variables included in the analyses were obtained from the provincial Thai Meteorological Department (TMD) from January 2002 to December 2018. They are (i) average monthly temperature (° C), (ii) average monthly relative humidity (%), (iii) average monthly rainfall (mm), (iv) average monthly number of rainy days (days), (v) average monthly wind speed (knot, where 1 knot = 1.334 km/h), (vi) average monthly evaporation (mm), (vii) average monthly cloud cover (okta), and (viii) average monthly sea-level pressure (hPa). These variables were chosen because they have been among the most relevant environmental variables in previous studies in the area mainly temperature, rainfall, cloud cover, and sea-level pressure (Abdulsalam et al., 2022).

### Data analysis

Since disease rates or mortality rates by age group were not available for all 23 districts (ideal for this type of study), an alternative indicator was generated from the cumulative number of cases observed in each year and the expected cases. This pseudo-value of incidence rate is simply referred to as the incidence rate indicator (*p-SIR)* in this study and was calculated using the formula below, which emulates the standardized incidence rate formula:

\begin{eqnarray*}p-SIR= \frac{\sum _{j=1}^{23}{d}_{j}}{\sum _{j=1}^{23}{n}_{j}\ast {t}_{j}} \end{eqnarray*}

Where *d*_*j*_ denotes the number of cases observed in the *j*-th year, *n*_*j*_ is the size of the population of the district concerned, *t*_*j*_ represents the rate of occurrence of all districts in the *j*-th year, obtained from the relation:

\begin{eqnarray*}{t}_{j}= \frac{{C}_{j}}{{N}_{j}} \end{eqnarray*}

*C*_*j*_ is the number of cases in the *j*-th year of all districts, and *N*_*j*_ is the size of the population in that year, consequently, the product of *n*_*j*_∗*t*_*j*_ expresses the expected value in the *j*-th year.

Assuming the spatio-temporal data follow a stochastic process (Blangiardo & Cameletti, 2015) from an indexed sequence whose form is: $Y(s,t)= \left\{ y(s,t){|}(s,t)\in {{!}}^{2}\ast {!} \right\} $ where *y* (*s*, *t*) represents the observed number of dengue cases in district *s* between 1 and 23 districts, at a time *t* between 1 and 17 years (2002–2018). If the data follow a Poisson distribution, with mean (*λ*_*st*_), the standardized incidence rate in districts can be modelled from a Poisson distribution.

After calculating *p-SIR*, also referred to in the results section as incidence rate, the following analyses were performed:

(1) A preliminary analysis of the raw data was made, analysing their frequency distribution, observing their descriptive statistics and evaluating their behavioural pattern under a scenario of an absence of spatial and temporal auto-correlation.
(2) Next, the risk of dengue in the *s* district in the *j-th* year was modelled as a function of the eight environmental variables in a log-linear Poisson model (Lee, 2020). This was done by maximum likelihood estimation using the *glm* function of R by choosing the Poisson family (R Development Core Team, 2021). The null hypothesis of residual spatial autocorrelation was verified with Moran’s statistic(Moran, 1950) with 10,000 random permutations. This statistic is a modified version of Pearson’s correlation coefficient whose values fluctuate between −1 and 1 (Earnest et al., 2007; Lee, 2020). Values close to 1 suggest spatial dependence, and values close to zero indicate spatial independence.
(3) Since preliminary analyses revealed that the incidence rate follows a fluctuating behaviour over time, similar to a wave function or Markov chain, in addition to observing signs of spatial autocorrelation, we opted to use a spatio-temporal autoregressive model with a Bayesian approach. For this, the R package CARBayesST was used following the suggestion of Rushworth, Lee & Mitchell (2014). CARBayesST package employs generalized linear mixed models of a spatio-temporal nature, whose inference is performed in a Bayesian setting by means of Markov chain Monte Carlo simulation Lee et al. (2021). The Markov chain Monte Carlo (MCMC) samples from independent Markov chains were generated using *ST.CARar* function from the same CARBayesST package. In this paper, we transcribe the initial expressions of the referred model. The complete formulas from their first reasoning, the precision matrix, the neighbourhood matrix, the explanation of the control of the spatial and temporal autocorrelation levels are mentioned in Leroux, Lei & Breslow (2000), Lee (2020), Lee et al. (2015), Lee et al. (2021) and works cited in these papers. According to Lee (2020), the referred model is given by *ψ*_*t*_ = *ρ*_*T*_*ψ*_*t*−1_ + *ϵ*_*t*_. Where ${\psi }_{t}= \left( {\psi }_{1t,\ldots \ldots \ldots ,}{\psi }_{Kt} \right) $ denotes the vector of random effects for all areal units at time *t* , and the vector of errors ${\epsilon }_{t}= \left( {\epsilon }_{1t,\ldots ..,}{\epsilon }_{Kt} \right) $ is modelled as spatially autocorrelated. Temporal autocorrelation is controlled by the mean function *ρ*_*T*_*ψ*_*t*−1_,  while spatial autocorrelation is controlled by the covariance structure of *ϵ*_*t*_. The latter is modelled as spatially autocorrelated and given by ${\epsilon }_{t}N \left( 0,{\tau }^{2}Q{ \left( W,{\rho }_{S} \right) }^{-1} \right) $, where *τ*^2^ is the process variance. To assess whether the Markov chains converged in terms of parameter estimates, a *trace plot* of the sample was drawn for the significant covariates. Each chain was run for 3,000,000 samples, of which 250,000 were removed during the burn-in period and the remaining 2,750,000 samples were thinned by 1,000 to remove correlation amongst the samples. If the samples showed no trend in their means or variances, it indicated convergence. To establish MCMC convergence, we used the Gelman–Rubin convergence diagnostic, which provides a summary of numerical convergence based on multiple chains, a value less than 1.1 indicates a good mixing of the chain (Gelman et al., 2013). These diagnostic checks were done for a sample of the spatio-temporal random effects $ \left\{ {\psi }_{kt} \right\} $ and for $ \left( \beta ,{\tau }^{2},{\rho }_{S},{\rho }_{T} \right) $ as suggested by Lee (2020).
(4) Considering spatial and temporal auto-correlation, we wanted to know to what extent environmental factors affect the risk of dengue infection in the study area. The effect of the covariates on dengue risk was quantified in relative terms by looking at the mean response of dengue risk to the fixed increase *ξ* in the value of each covariate. For each covariate, the increase *ξ* is the standard deviation as it better reflects increases in the value of the covariate and to compute the estimated relative risks, we first construct a matrix of the MCMC samples for the regression parameters $ \left( {\beta }_{1},{\beta }_{2} \right) $ from all chains, replicating the suggestion of Lee (2020).
(5) Finally, we choose to use the interquartile range (IQR) to illustrate the total health inequality (WHO, 2013) related to dengue infectious disease, which measures the variation of the disease risk estimates over the study region within the study period. IQR variation was quantified separately for each year by measuring the difference between the third and first quartiles of dengue risks for a given year.

### Ethical consideration

The Ethics Committee of Walailak University granted ethical approval for the study protocol with project number WU-EC-AH-0-226-63 and approval number WUEC-20-151-01/02. Data privacy and confidentiality were duly ensured and all information obtained was anonymized.

## Results

**Figure 2:**
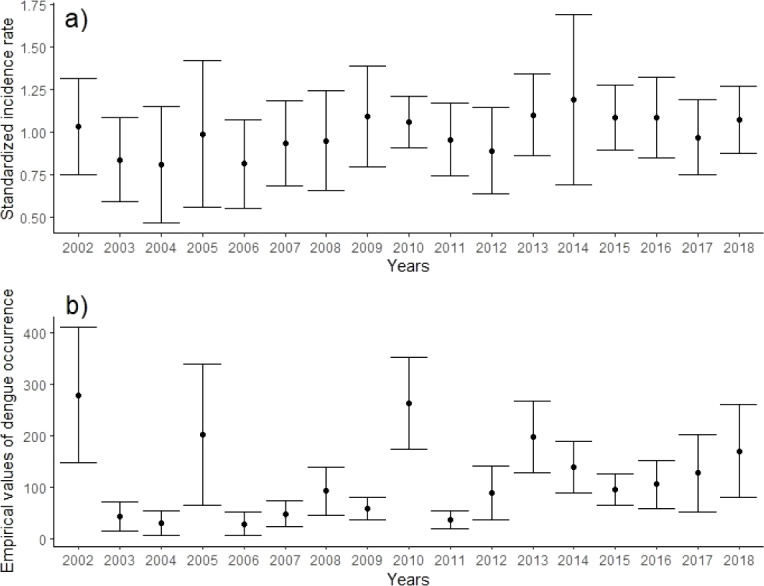
Trend of the dengue incidence rate showing (A) the observed values and (B) over time at the 95% confidence level.

The evolution of the incidence rate fluctuated over time, although the confidence intervals from one year to another overlapped, suggesting that despite the high variability, the probability of observing statistically different risk rates from one year to another was low (Fig. 2A, Figs. S1 and S2). Yet, this trend contrasts with the trend of the empirical occurrence, whose differences were evident between some years with respect to others (Fig. 2B) as in the case of 2002, 2005, and 2010 which were statistically higher than those of 2003, 2004 and 2011 (Fig. 2B).

**Figure 3:**
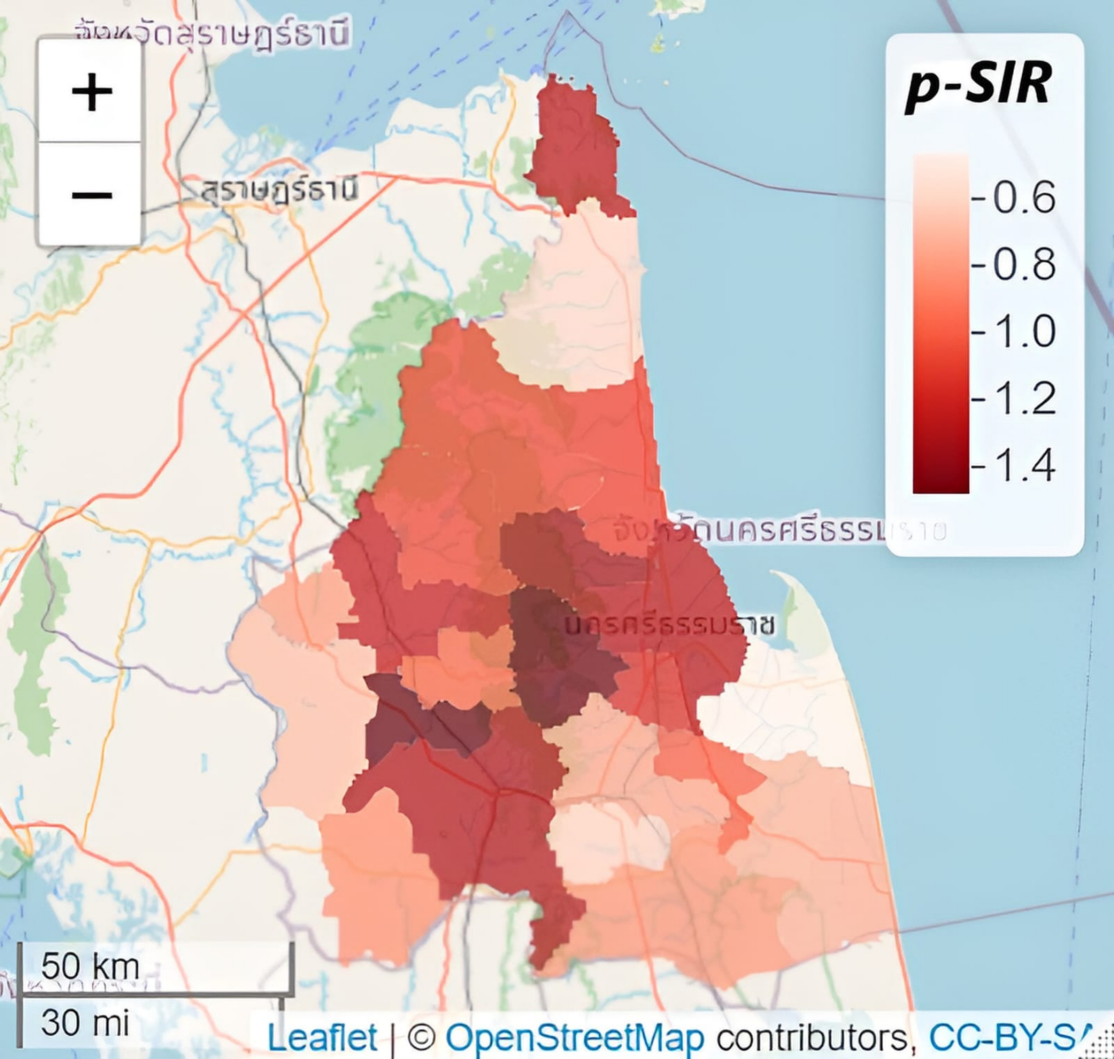
Average spatial pattern of the incidence rate indicator from 2002 to 2018. Map data: Leaflet —©OpenStreetMap contributions, CC BY SA.

On the other hand, at the district level, a variation of risks was observed from one to another. For example, Fig. 3 shows the average spatial pattern in the SIR and from the map, the highest risk areas seem to be the centrally located districts (*i.e.,* Lan Saka, Nabon, Chawang, Thung Song, Phrom Kili, and Mueang), and the lower risk areas are the south-eastern located districts (Churapon and Pak Phanang) and Sichon district. The higher-risk areas tend to be grouped together suggesting a spatial correlation in the data.

The degree of correlation between the covariates is observed in Fig. S3. In the correlogram, stronger colours suggest strong associations for pan evaporation, relative humidity (Rhumidity), and number of rainy days (NumRainyDays). Medium or low colours show a positive correlation between pan evaporation and temperature, sea-level pressure and wind speed (*r* is between 0.4 and 0.6). No correlation was observed between the covariates pan evaporation and cloud volume. After the Bonferroni correction, the point estimate of the weather covariates that exhibits significant effects on dengue incidence risk in the simple Poisson model, under a scenario of an absence of spatial and temporal auto-correlation, were temperature, number of rainy days, windspeed and sea-level pressure. According to the Poisson model, an increasing level of temperature, number of rainy days and sea-level pressure is associated with an increased risk of dengue incidence, while an increasing level of windspeed is a protective factor (Table 1). In the southern tropical coasts of Thailand, it has been reported that sea-level pressure has been a constant predictor of dengue incidence and outbreaks (Abdulsalam et al., 2021). Figure S3B is a scatter plot that shows the relationships between the average log of dengue cases and environmental variables.

**Table 1:** Parametric coefficients showing the fit indicators of the final model. The *p*-value is the value of the probability against the null hypothesis when testing the null significant contribution of each predictor in the regression model.

Variable (Average monthly)	Estimate	Lower limit (0.025)	Upper limit (0.975)	Std. Error	*z*-value	*p*-value	*p*-value after Bonferroni Correction
(Intercept)	−73.783	−103.533	−44.013	1.52E+01	−4.859	1.18E−06	0.00001
Temperature (° C)	0.055	0.011	0.100	2.27E−02	2.437	0.015	0.11840
Relative humidity (%)	−0.011	−0.025	0.003	7.07E−03	−1.591	0.112	0.89280
Rainfall (mm)	0.000	0.000	0.001	1.52E−04	1.664	0.096	0.76800
Number of rainy days (days)	0.013	0.001	0.026	6.48E−03	2.056	0.040	0.31840
Wind speed (knots)	−0.176	−0.202	−0.150	1.33E−02	−13.26	<2e−16	0.00000
Pan evaporation (mm)	0.001	−0.001	0.003	9.31E−04	0.772	0.440	3.52080
Cloud cover (okta)	−0.015	−0.048	0.018	1.69E−02	−0.883	0.377	3.01600
Sea-level pressure (hPa)	0.073	0.043	0.102	1.50E−02	4.827	1.39E−06	0.00001

Results to detect the presence of spatial autocorrelation were done for all years. However, by way of illustration, we used the one for the year 2014 (see Table S1). The test for the year 2014 shows sufficient evidence of spatial autocorrelation of the residuals, with a Moran’s I value of 0.5539 and a *p*-value much less than 0.05 (Table S1). Observing the MCMC simulations, results show no change in the mean or variance of the samples, as the chains appear to converge in Fig. S4 and (just below Fig. S4 is) the Gelman–Rubin diagnostic value of 1.01 indicating that the sample is well mixed and we can proceed with inference from this model. The results of our fitted algorithm show that all environmental parameters include 0 and; *τ*^2^ shows the random effect variation value of ∼0.73, *ρ*_*S*_ suggests a weak spatial correlation value of ∼0.32, and *ρ*_*T*_ shows a moderate temporal correlation value of ∼0.45 (see Table S2). The Geweke diagnostic is essentially a *z*-score value of ±1.96 suggesting a convergence in our data as observed (see Table S2). For all years, the same analyses were done.

Regarding the effects of covariates on the risks of occurrence, the highest a posteriori relative risk medians were observed for pan evaporation with a value of 1.014, followed by sea-level pressure with 1.013, rainfall and number of rainy days with a value of 1.0 (Table 2). Although the 95% credible interval contains the null risk of 1, for all variables, the relative values reveal that if pan evaporation increases by 12.273 mm, the risk of dengue increases by 1.4% and when sea-level pressure increases by 0.432 hPa, a risk increase of 1.3% would be expected (Table 2).

**Table 2:** Medians of the a posteriori relative risk considering a 95% credible interval and the fixed increase of each covariate.

**Covariates**	**Fixed increase of each covariate (*ξ* )**	**Median**	**2.50%**	**97.50%**
Temperature (° C)	0.351	0.998	0.873	1.132
Relative humidity (%)	1.502	0.973	0.767	1.217
Rainfall (mm)	45.541	1.000	0.878	1.140
Number of rainy days (days)	1.261	1.000	0.853	1.170
Wind speed (knots)	0.697	0.977	0.809	1.174
Pan evaporation (mm)	12.273	1.014	0.811	1.269
Cloud cover (okta)	0.436	0.969	0.821	1.143
Sea-level pressure (hPa)	0.432	1.013	0.877	1.169

**Figure 4:**
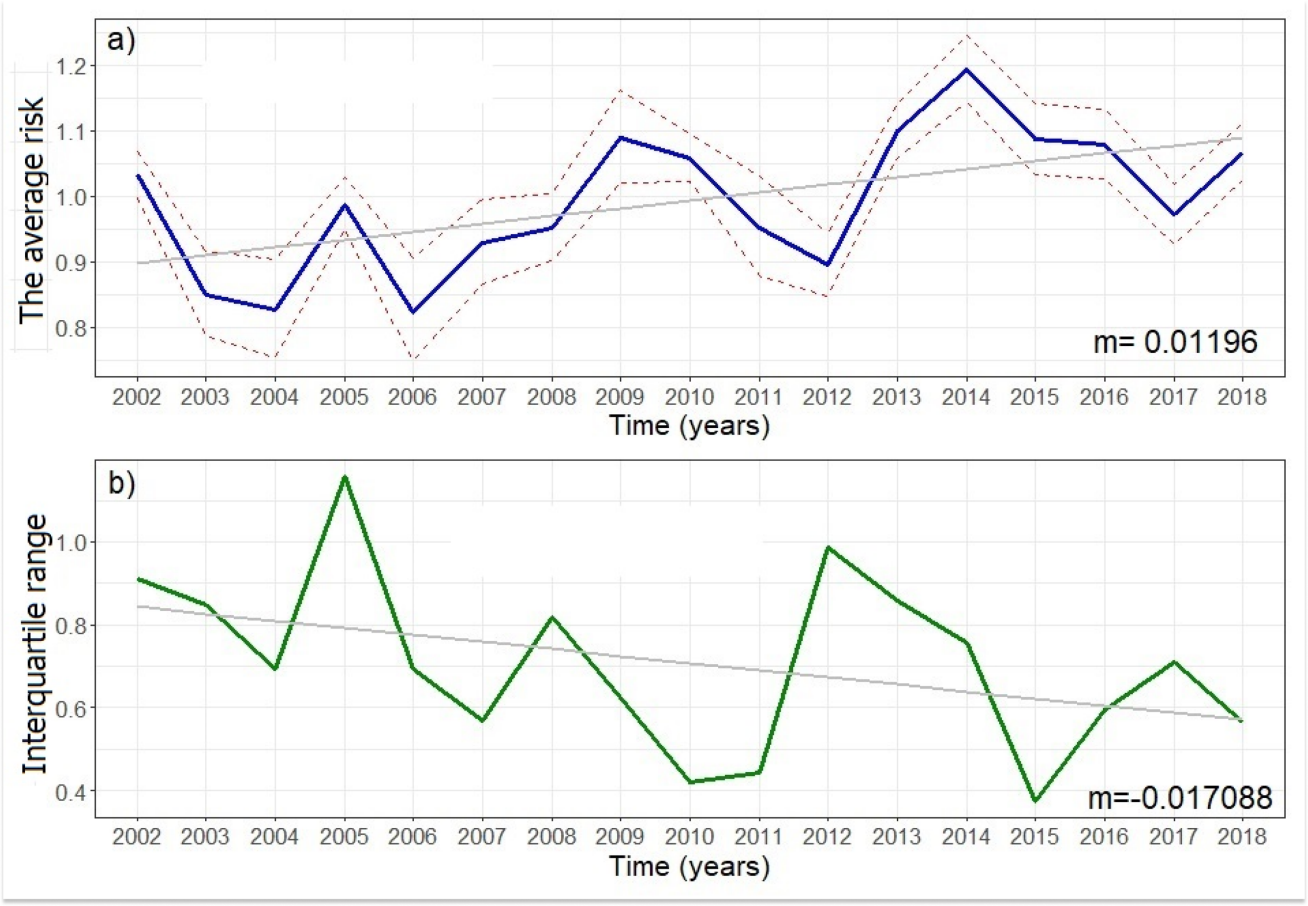
The posterior median (solid blue line) and 95% confidence interval (dash lines) for the temporal trend in disease risk (A) and estimated temporal trend (B) (solid green line) in the health inequality in dengue risk as measured by the spatial interquartile ran.

For our concern regarding the posterior risk distributions for each year across the districts and the spatio-temporal trend in disease risk, the estimated average temporal trend shows an upward trend and peaks every 4–5 years as shown below (Fig. 4). Similar to what was observed in the exploratory analysis, both the relative risk and the disease inequality values showed fluctuating trends considering short time periods. For example, from 2002 to 2004, and from 2014 to 2017 a decreasing trend was observed; on the contrary, from 2006 to 2009, and from 2012 to 2014 increasing trends were observed (Figs. 4A and 4B). However, considering the entire 17-year period studied, an average rate of change of 0.012 of the relative risk for each year elapsed was observed (Fig. 4A); whereas, for the inequality values, it was negative, being −0.017 for each year elapsed (Fig. 4B).

**Figure 5:**
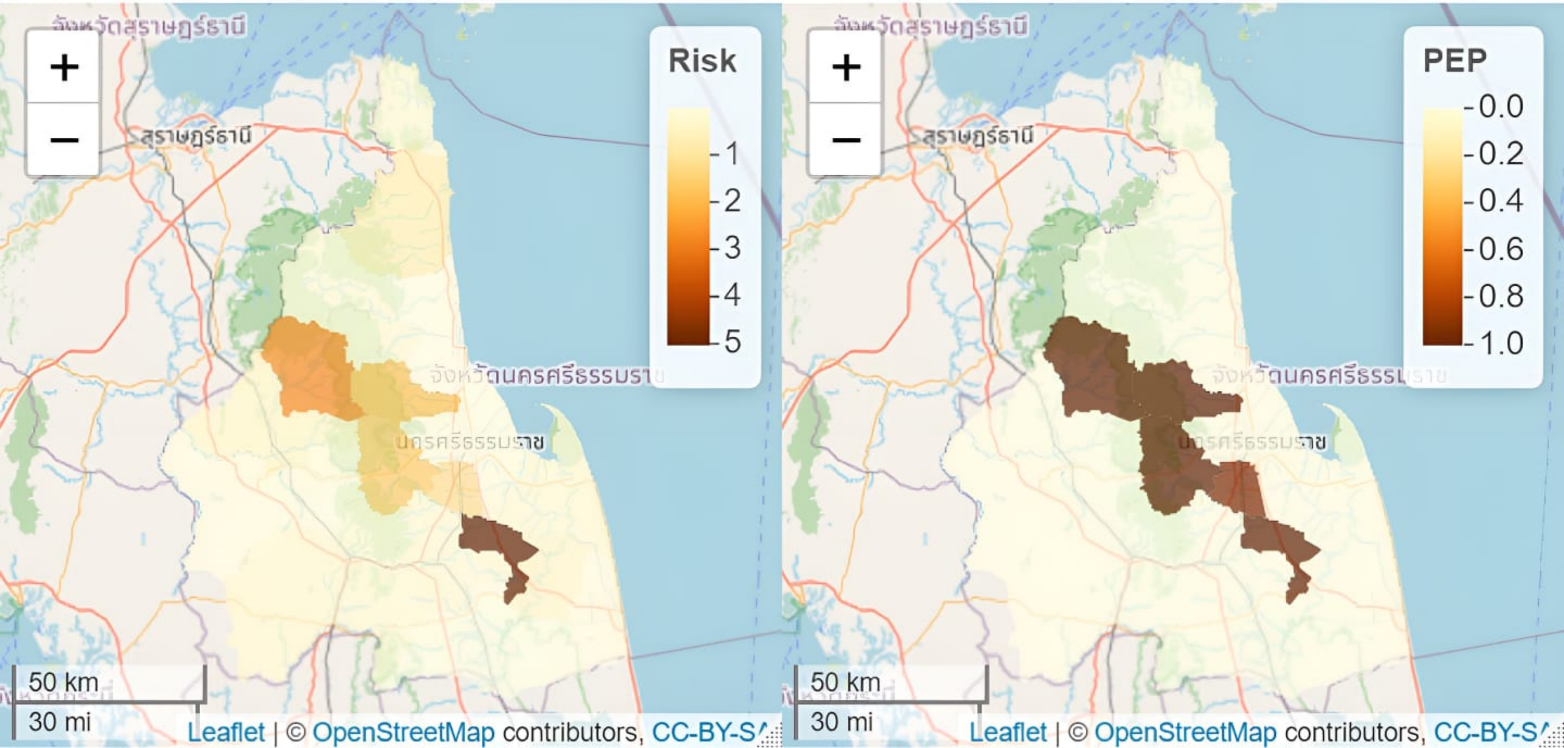
Estimated (posterior median) risk surface for 2014 (left) and the posterior exceedance probabilities showing that risk in 2014 is greater than 1 (right). Map data: Leaflet —©OpenStreetMap contributors, CC BY SA.

A map of the spatial risk pattern can be done for all years but in this study, it was illustrated only for the year 2014 (Fig. 5). The spatial pattern in risk is displayed in two ways *i.e.,* the posterior median risk surface estimates ${\hat {\theta }}_{kt}$ and the posterior exceedance probability (PEP) which shows the probability that the risk in an area exceeds 1. Both quantities (median risk and PEP) are illustrated below in Fig. 5 showing a similar pattern spatially to our SIR map (Fig. 3). It is observed to be smoother as we assume to have spatial correlation in our data, the highest risk areas pan across diagonally from the north-western districts to the south-eastern districts (*i.e.,* from Phipun, Phrom Kili, Lan Saka, Phra Phrom and Chaloem Phakiat).

PEP also shows the probability of having an elevated risk. Most of the districts are light-coloured and have no probability of exceeding the average risk of 1, while the dark areas have the probability of exceeding a null risk of 1 (district names from left to right are Phipun, Phrom Kili, Lan Saka, Phra Phrom and Chaloem Phakiat).

**Figure 6:**
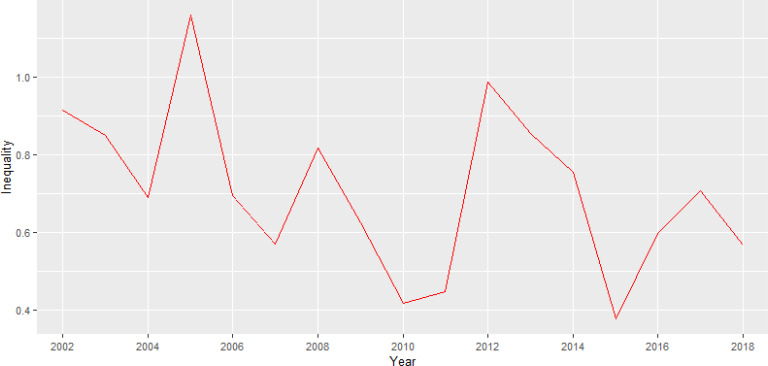
Estimated temporal trend in the health inequality in dengue incidence risk as measured by the spatial interquartile range.

Lastly, on the question about health inequality, we look at the range in variation of the estimated dengue risk across all 23 districts using the IQR (interquartile range). Figure 6 below displays the temporal trend in IQRs and the overall trend in inequality seems to decline from a value of ∼0.9 in 2002 to ∼0.6 in 2018. The total inequality in dengue incidence risk measured is narrowing and this suggests an increasing population even in terms of risks in later years.

## Discussion

The study illustrates a district-level estimation of dengue risk in both space and time, drawing inferences to answer epidemiologically important questions. It is important to understand how the interactions between climatic factors over past decades have defined infection trends and risks, unique to every region. Results of previous studies have shown that mean annual temperature, sea level pressure, number of days of rainfall and wind speed are among the variables having a significant influence on the rate of occurrence of dengue fever in the study area (Abdulsalam et al., 2022), which was confirmed in this study (Table 1). In coastal areas, temperature and precipitation tend to be higher and the study region is located in a coastal region (the Gulf of Thailand). While there may not be a substantial change in geographical patterns over time, there is a visible increase in precipitation in the region for the past decade (Abdulsalam et al., 2022) and from April to October, increasing temperature and precipitation levels also increase mosquito density ultimately driving up the number of reported dengue cases. The temporal trend in dengue incidence risk observed in Fig. 2 shows a fluctuating pattern of dengue infection over the years. The outliers may be instances of epidemic occurrence as, from the year 2012, reported dengue incidence in the province has continued to be above the threshold during monsoon seasons. Increasing annually recorded number of rainy days and windspeed in this study have a negative correspondence with disease risk. Although rainfall favours the proliferation of mosquitoes, in excessive amounts, it disrupts the mosquito vector life cycle flushing out the aquatic phase from breeding sites, also, strong winds tend to reduce mosquito-biting opportunities making it difficult to find a host (Reid, 2000). Figure 3 presents the *p-SIR* from 2002–2018 of all districts ranging from 0.5 to 1.5. Influenced by weather, environment and humans as risk factors, the variability of dengue incidence across space and time can be observed in the figure showing a spatiotemporal pattern with some centrally located districts having a *p-SIR* greater than one during the time period similar to a recent study where high-risk clusters were located in the north, central and southern districts (Jaya & Folmer, 2021). Due to sparsely available data for other determinants, we focused on weather variables obtained from the Thailand Meteorological Bureau considering all variables generally regarded as important dengue disease risk factors (Abdulsalam et al., 2022; Zellweger et al., 2017).

In local epidemiological dynamics, precision may be lost in evaluating disease risk over broad scales of space and time as done in the past. For instance, within the study period, Lan Saka and Mueang districts are known to be more consistent hotspots for dengue fever incidence compared to their surrounding districts (Abdulsalam et al., 2021) and to incorporate local dynamics of disease circulation, they should be noted as examples of the need for public health modelling. Our models identified the boundaries having high-risk differentials with their surrounding areal units as Fig. 5 shows the spatial pattern of average relative risk showing localization of higher risk areas from the north-western to the south-eastern districts of the region similar to a study (Puggioni et al., 2020).

The upward trend in spatio-temporal disease risk over the years shows the need to address dengue issues of local epidemiological anomalies and the support for precise public health decision-making. In the future, the probability of having an elevated risk of dengue applies to Phipun, Phrom Kili, Lan Saka, Phra Phrom and Chaloem Phakiat districts (Fig. 5). The higher risk in these districts could be attributed to the topography, which is mountainous in nature and have agricultural plantations of rubber, coconut and oil palm commercialisation making it suitable for mosquito breeding. Depending on human activity, climate conditions and physical geography (Liu et al., 2012), each mosquito species has its preferred breeding sites for oviposition (Muhammad-Aidil et al., 2015). To mention but a few, breeding sites can be natural or artificial, permanent or temporary, shaded or sunny and are found in running or stagnant water bodies. A report has shown that coconut shells are preferred breeding sites often chosen by mosquitoes and eggs of *Aedes spp* are usually found in such habitats (Rao et al., 2011). Since most of the coconut fruit is gnawed open by rodents after falling from its tree or its shells are improperly discarded after use, they end up having a small orifice and are dark inside making it an ideal breeding ground for *Aedes* mosquitoes. Also, districts with rubber plantations (including the aforementioned districts) (National Statistical Office, 2015) expose those living within the area to day-biting mosquitoes as they are potential breeding sites for *Aedes spp* (Abdulsalam et al., 2021). According to Messina et al. (2019), it is expected that the population at the greatest risk of dengue infection will continue to grow substantially and disproportionately, particularly in the most economically disadvantaged areas like Chaloem Phakiat, Chang Klang and Than Phanra.

Dengue morbidity and mortality can be affected by socioeconomic deprivation which is one of the main drivers of health inequalities (WHO, 2013). The total inequality in dengue incidence risk measured in our study is narrowing indicating a decline (Fig. 4B and Fig. 6). The incidence of dengue infection has been related to low income, education, inadequate sanitation and water supply in northern Brazil (Da Conceição Araújo et al., 2020). The results of health inequality monitoring have an impact on informed practices, programmes and policies as policymakers are increasingly looking to quantitative evidence. Addressing health inequalities especially at the district level leads to a better national health system.

In modelling dengue fever risk, the three key foci of information that are used are climate factors and environmental suitability, disease circulation and entomological data; and of all three, the most difficult and costly to collect is entomological data. This is to say that in the absence of vector abundance data, modelling approaches that achieve precisely a level of disease risk prediction informatory for public health decision-making are needed. A study based on a time-series analysis reporting the distinguishing effects of precipitation and temperature on dengue incidence from mosquito-mediated impacts found the strongest correlation and significant positive association to being between precipitation and outbreak intensity, and temperature and outbreak risk (Xu et al., 2017). This suggests that in predicting the variability in dengue incidence, modelling weather in the absence of mosquito population data may be sufficient as the role of weather in dengue transmission cycles is mediated by its impact on mosquito survival.

Although vector population data is not included in the models, this approach inherently acknowledges the importance of mosquito vector biology impacted by environmental factors as, temperature plays a role in mosquito development time (Becker et al., 2010; Couret, Dotson & Benedict, 2014), extrinsic incubation period of dengue virus (Chan & Johansson, 2012) and vectorial capacity (such as survival and bite rate) (Yasuno & Tonn, 1970; Liu-Helmersson et al., 2014). In Thailand, discarded water containers, tires, coconut shells, and potted plants are common habitats positively correlated with mosquito density (Harrington et al., 2008; Haddawy et al., 2019), hence the need for further studies to determine the extent to which dengue disease risk estimates using this modelling approach can be improved by including entomological data. In addition, in districts where multiple pathogens circulate, the extent to which coinfection in mosquitoes may limit or interfere with vectorial capacity is unknown and merits further studies.

Dengue fever continues to be a vector disease problem and spatio-temporal analysis can be a valuable tool for disease risk estimates. Weather variables have important future implications for epidemiological studies of mosquito-borne diseases particularly at the district level as unique local factors of a province cannot be generalized to other provinces, regions or even at the national level. For strategic planning of intervention measures, dengue prediction models should be determined at a small scale, valuable to the Ministry of Public Health. Findings reported here constitute an advance in temporal and spatial knowledge of dengue in the Nakhon Si Thammarat region, helpful to decision-makers. Finally, we recognize that the results reported here could be further strengthened by additional studies in the future. For example, including the mortality rate, cases by age, and other environmental variables in the database would be advantageous. Furthermore, complementary analyses such as sensitivity analysis (Depaoli, Winter & Visser, 2020), Bayesian predictive interval analysis (Dybowski & Roberts, 2001; Hamada et al., 2004), and Robust Bayesian analysis (Berger et al., 1994) could be implemented to enhance the results.

## Conclusion and Policy Implication

From the study, several conclusions emerge as it illustrates how spatiotemporal areal unit data can be analysed by employing the CARBayesST package in R, fitting models in a Bayesian setting *via* MCMC simulation, subsequently answering important epidemiological questions. It further confirms that in the role of dengue disease transmission, temperature, number of rainy days, windspeed and sea-level pressure are determining factors at the district level. In addition, the framework presented is appropriate for an early warning and response system as it allows for the prediction of dengue outbreaks per district; and this can also be applied to other mosquito-borne disease problems.

The study allows insight into the local contextualization of the dengue fever problem providing evidence of the potential probability of having elevated dengue incidence risks in some districts in the southern region of Thailand. Considering the results obtained in this study, particularly the maps, the need to obtain scale-level maps is evident. Thus, the Ministry of Public Health could use these results for strategic planning of intervention measures. In the event of near future, the threat of elevated disease risk needs to be prevented and controlled considering the factors underlying the spread of mosquitoes in the Southeast Asian region.

##  Supplemental Information

10.7717/peerj.15619/supp-1 Supplemental Information 1 Supplemental Figures and Tables

10.7717/peerj.15619/supp-2 Supplemental Information 2 Raw data

## Additional Information and Declarations

### Data Availability

The following information was supplied regarding data availability: The raw measurements are available in the Supplemental Files.
