# Supplementary material for: Spatio-temporal dengue risk modelling in the south of Thailand: a Bayesian approach to dengue vulnerability
Source: PeerJ. 2023 Jul 14;11:e15619. doi: 10.7717/peerj.15619 (PMC10351518; doi:10.7717/peerj.15619)
Supplement: Supplemental Information 1 [file peerj-11-15619-s001.docx]

Additional file 1.

| 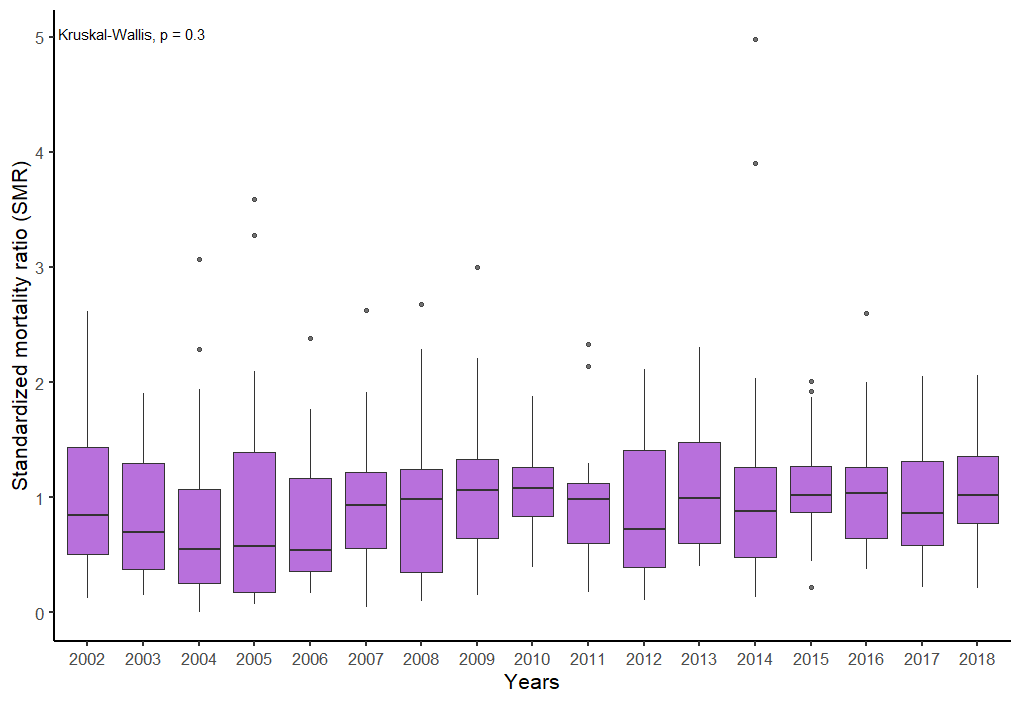 |
| --- |
| **Figure S1.** The mean time trend of dengue risk 2002-2018. |

| 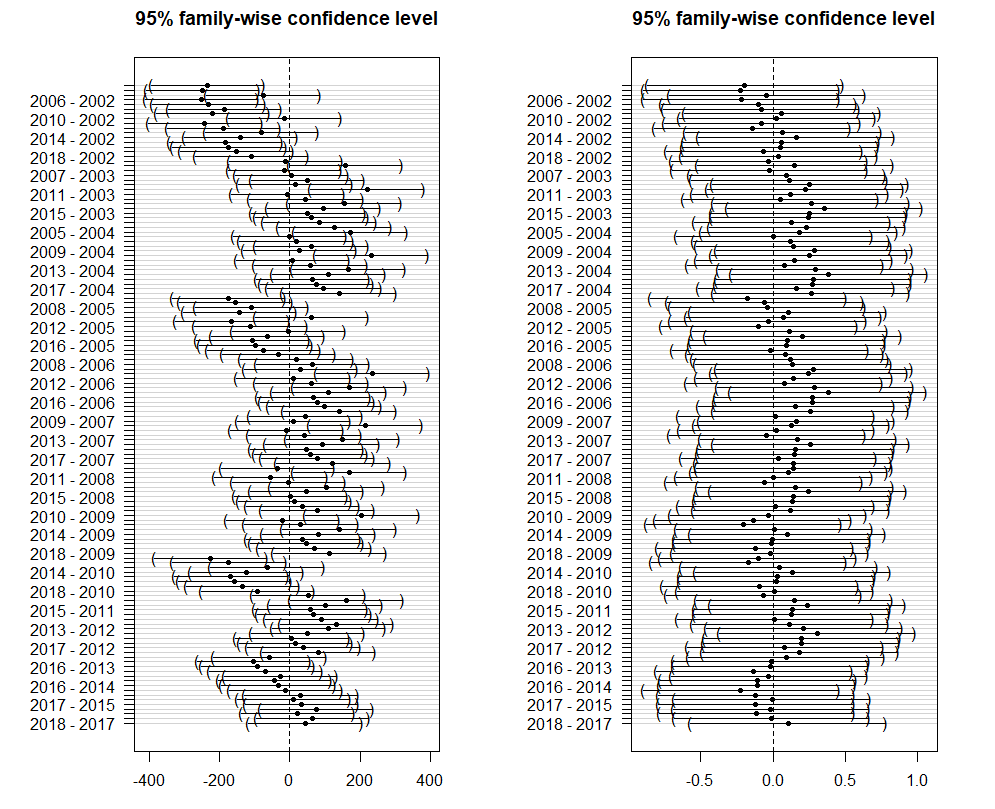 |
| --- |

**Figure S2.** Average rate of change with their respective confidence intervals (95%) for the empirically reported dengue cases (A) and the incidence rate (B)

| 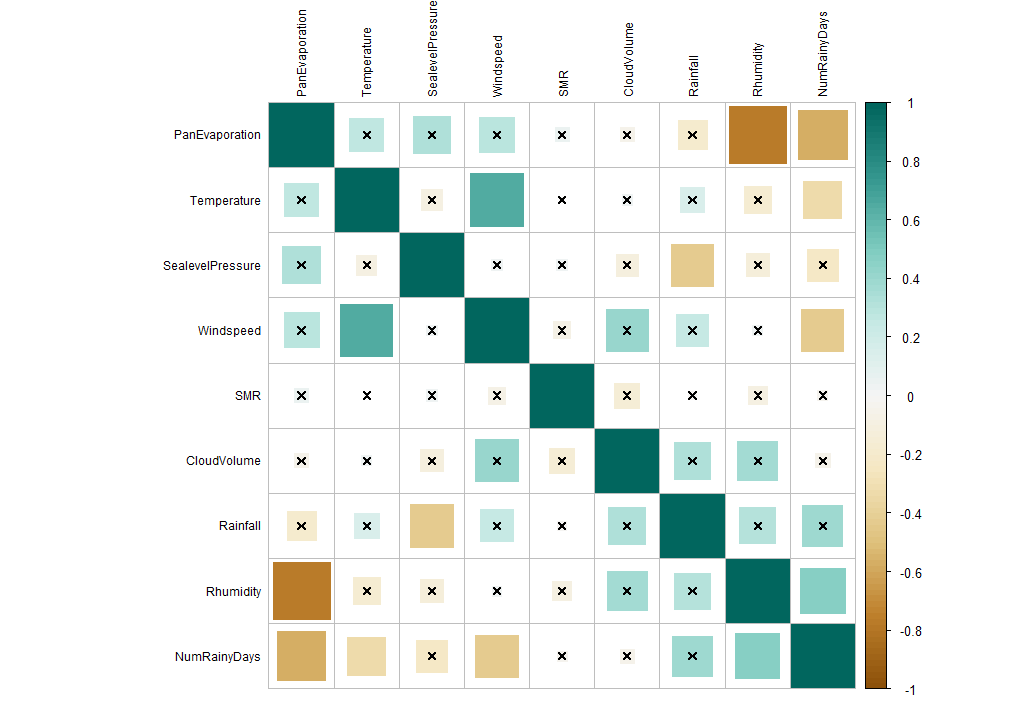 |
| --- |
| **Figure S3a** Correlation matrix between incidence rate and the eight environmental variables. |

| 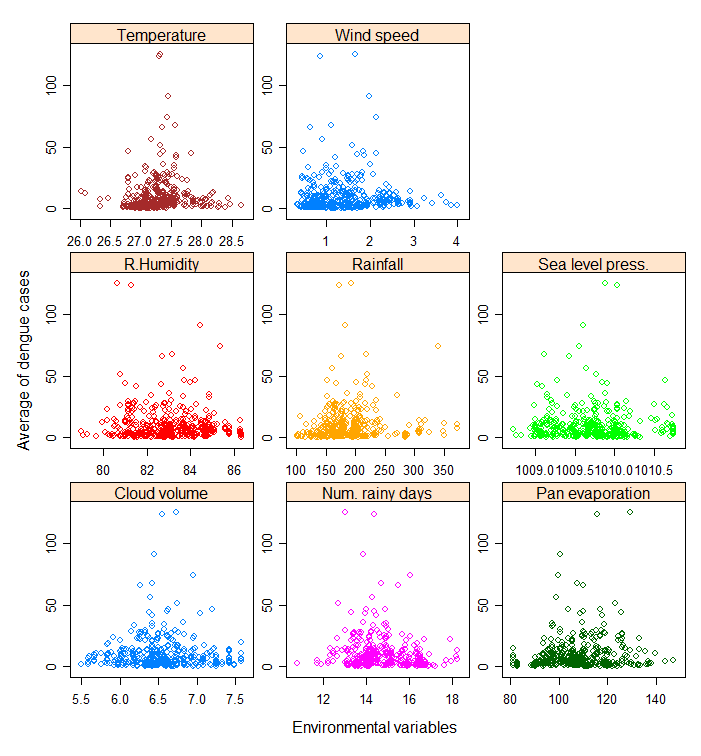 |
| --- |
| **Figure S3b** Scatter plot showing the relationships between the average log of dengue cases and environmental variables. |

| 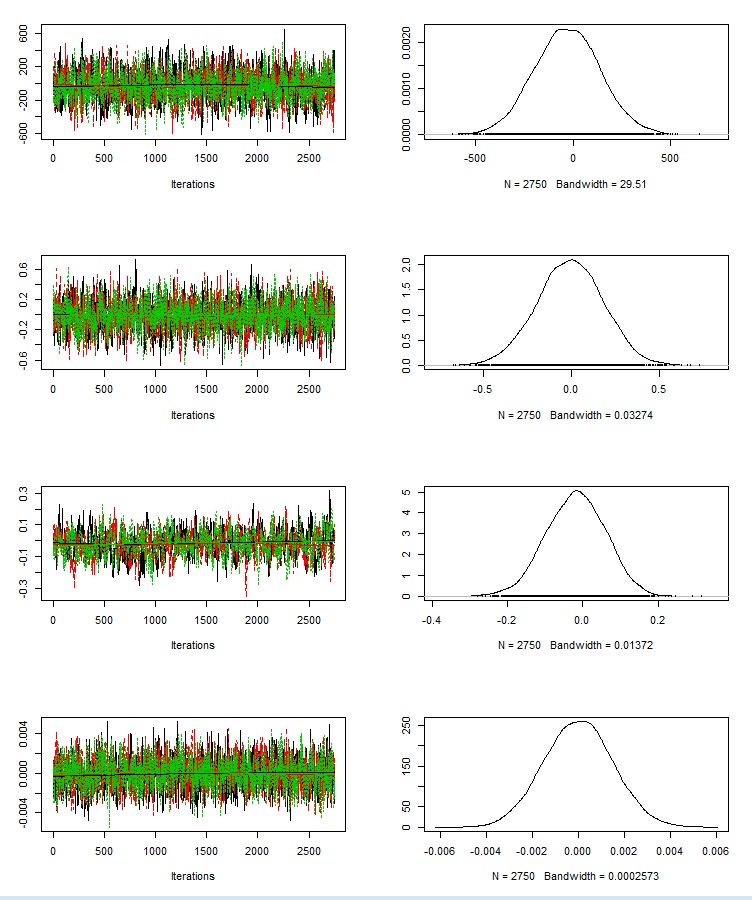  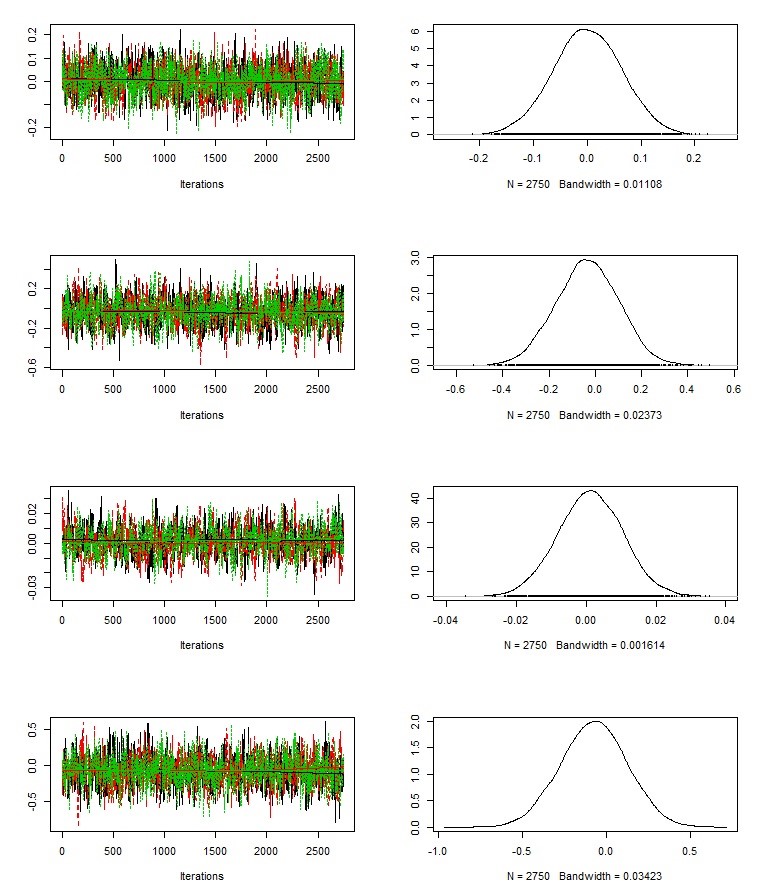  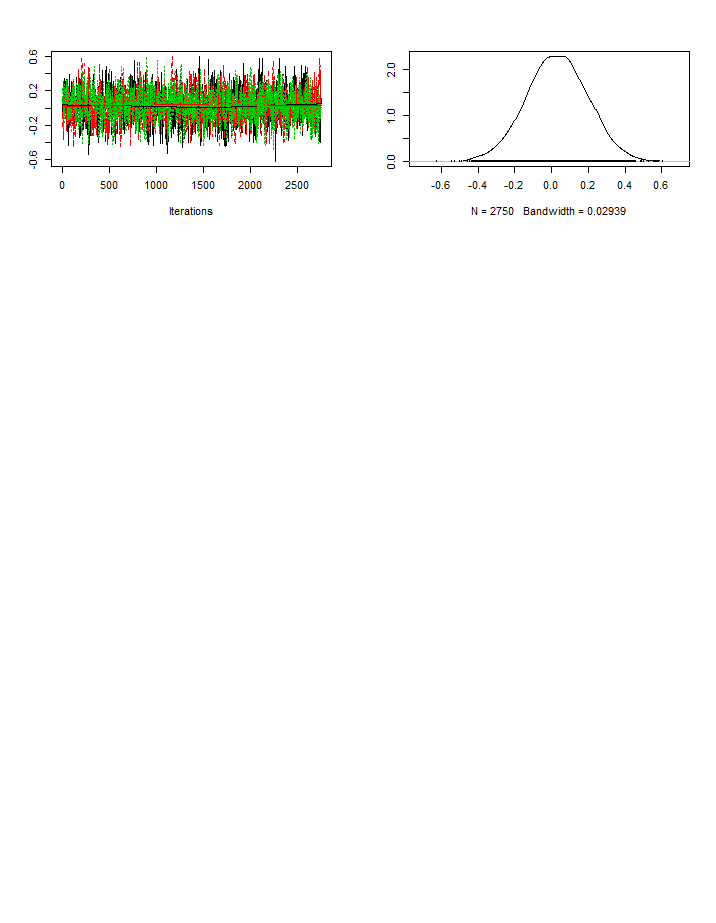 |
| --- |
| **Figure S4**. Traceplots generated for the MCMC samples. |

**Table S1.** Table S1 Moran's statistic results. Highlighted in bold are years with evidence of spatial correlation (alpha=0.05).

| Year | Moran's statistic | P-value |
| --- | --- | --- |
| 2002 | 0.075764 | 0.168300 |
| **2003** | **0.16579** | **0.051290** |
| 2004 | -0.056199 | 0.504600 |
| **2005** | **0.3487** | **0.001500** |
| 2006 | -0.13231 | 0.764100 |
| 2007 | -0.19826 | 0.908900 |
| 2008 | -0.13231 | 0.757300 |
| 2009 | -0.20893 | 0.916100 |
| 2010 | -0.0085479 | 0.361400 |
| **2011** | **0.15404** | **0.045900** |
| **2012** | **0.32341** | **0.003400** |
| 2013 | 0.071003 | 0.170000 |
| **2014** | **0.5539** | **0.000100** |
| 2015 | -0.13728 | 0.782600 |
| 2016 | -0.091715 | 0.626700 |
| **2017** | **0.24017** | **0.012400** |
| 2018 | 0.11433 | 0.074690 |

Potential scale reduction factors:

Point est. Upper C.I.

[1,] 1.00 1.00

[2,] 1.00 1.00

[3,] 1.00 1.02

[4,] 1.00 1.00

[5,] 1.00 1.00

[6,] 1.00 1.00

[7,] 1.01 1.02

[8,] 1.00 1.00

[9,] 1.00 1.00

Multivariate psrf

1.01

**Table S2**. Parameters of the likelihood model–Poisson for independent Markov chains.

|  | Median | 2.50% | 97.50% | n.effective | Geweke.diag |
| --- | --- | --- | --- | --- | --- |
| First run | | | | | |
| (Intercept) | -22.0315 | -366.0566 | 330.3484 | 489.5 | 0.2 |
| Temperature | -0.0068 | -0.3936 | 0.346 | 692 | -0.1 |
| Relative humidity | -0.0147 | -0.1776 | 0.1425 | 186.4 | 0.5 |
| Rainfall | 0 | -0.003 | 0.0029 | 727.8 | -1.9 |
| Number of Rainy Days | -0.0005 | -0.1234 | 0.1254 | 424.6 | 0.8 |
| Windspeed | -0.0371 | -0.303 | 0.2256 | 361 | 0.5 |
| Pan Evaporation | 0.002 | -0.0163 | 0.0196 | 225.5 | 0.9 |
| Cloud Volume | -0.0771 | -0.4699 | 0.322 | 447.5 | 0.1 |
| Sea-level Pressure | 0.0225 | -0.3261 | 0.3676 | 472.6 | -0.2 |
| tau2 | 0.7336 | 0.5628 | 0.9501 | 2197.3 | -1.2 |
| rho.S | 0.3149 | 0.1746 | 0.4917 | 2159.5 | 0.5 |
| rho.T | 0.4529 | 0.3446 | 0.5578 | 1243.6 | -0.7 |
| DIC = 3075.107 p.d = 363.6948 LMPL = -1663.743 | | | | | |
| Second run | | | | | |
| (Intercept) | -32.2116 | -373.4226 | 298.5061 | 484.4 | -0.8 |
| Temperature | -0.0057 | -0.397 | 0.3521 | 610.1 | 0.4 |
| Relative humidity | -0.0224 | -0.1807 | 0.121 | 188.6 | -0.4 |
| Rainfall | 0 | -0.0028 | 0.0029 | 585.3 | -0.4 |
| Number of Rainy Days | -0.0001 | -0.1242 | 0.1252 | 382.9 | 1.9 |
| Windspeed | -0.0307 | -0.3069 | 0.2299 | 355.2 | 0.4 |
| Pan Evaporation | 0.0002 | -0.0182 | 0.0198 | 209.1 | 0.2 |
| Cloud Volume | -0.0677 | -0.4418 | 0.2981 | 457.4 | 0.4 |
| Sea-level Pressure | 0.0334 | -0.2917 | 0.3778 | 470 | 0.8 |
| tau2 | 0.7281 | 0.5618 | 0.947 | 2388.5 | 2.2 |
| rho.S | 0.3146 | 0.1786 | 0.4898 | 2429.7 | 0.8 |
| rho.T | 0.4533 | 0.3445 | 0.5559 | 1744.5 | 0.1 |
| DIC = 3074.287 p.d = 363.1903 LMPL = -1660.585 | | | | | |
| Third run | | | | | |
| (Intercept) | -32.8692 | -335.8036 | 286.1218 | 562.2 | -2 |
| Temperature | -0.0094 | -0.3549 | 0.3627 | 694 | -0.2 |
| Relative humidity | -0.0166 | -0.1713 | 0.1249 | 199.1 | -0.7 |
| Rainfall | 0 | -0.0027 | 0.0029 | 660.3 | 0.3 |
| Number of Rainy Days | 0.0012 | -0.1297 | 0.1191 | 495.7 | -0.2 |
| Windspeed | -0.0337 | -0.2968 | 0.2322 | 356.7 | -0.2 |
| Pan Evaporation | 0.0015 | -0.0163 | 0.0187 | 254.5 | -1 |
| Cloud Volume | -0.0689 | -0.4445 | 0.2965 | 370.3 | 0.4 |
| Sea-level Pressure | 0.0354 | -0.2849 | 0.3346 | 547.8 | 2 |
| tau2 | 0.7257 | 0.5736 | 0.9497 | 1734.6 | -0.3 |
| rho.S | 0.3137 | 0.1776 | 0.4894 | 1974.9 | -0.2 |
| rho.T | 0.4527 | 0.3482 | 0.5529 | 2108 | -0.3 |
| DIC = 3075.756 p.d = 364.0411 LMPL = -1669.427 | | | | | |
